# Supplementary figures and images for: Dental follicle mesenchymal stem cells ameliorated glandular dysfunction in Sjögren’s syndrome murine model
Source: PLoS One. 2022 May 5;17(5):e0266137. doi: 10.1371/journal.pone.0266137 (PMC9070867; doi:10.1371/journal.pone.0266137)

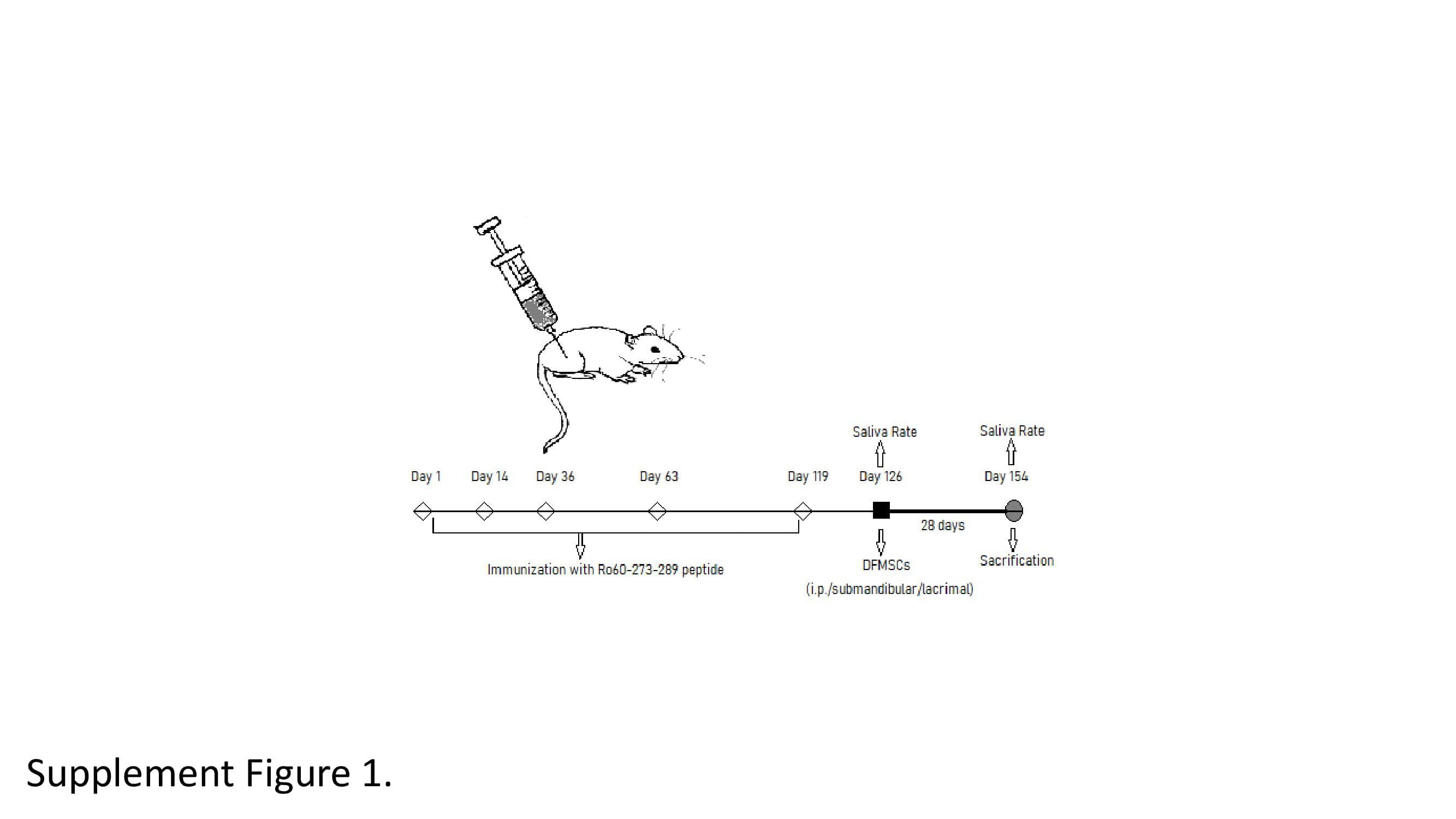

Supplement: S1 Fig — The SS murine model was performed with the intraperitoneal injection of 50 μg of Ro60 peptide emulsified in 100 μL of Freuds’ complete adjuvant (FCA) on day 1. The following immunizations were carried out on days 14, 36, 63, and 119 with 50 μg Ro60 peptide. DFMSCs are administered by intraperitoneal, submandibular, or lacrimal injections. (TIF) [file pone.0266137.s001.tif]

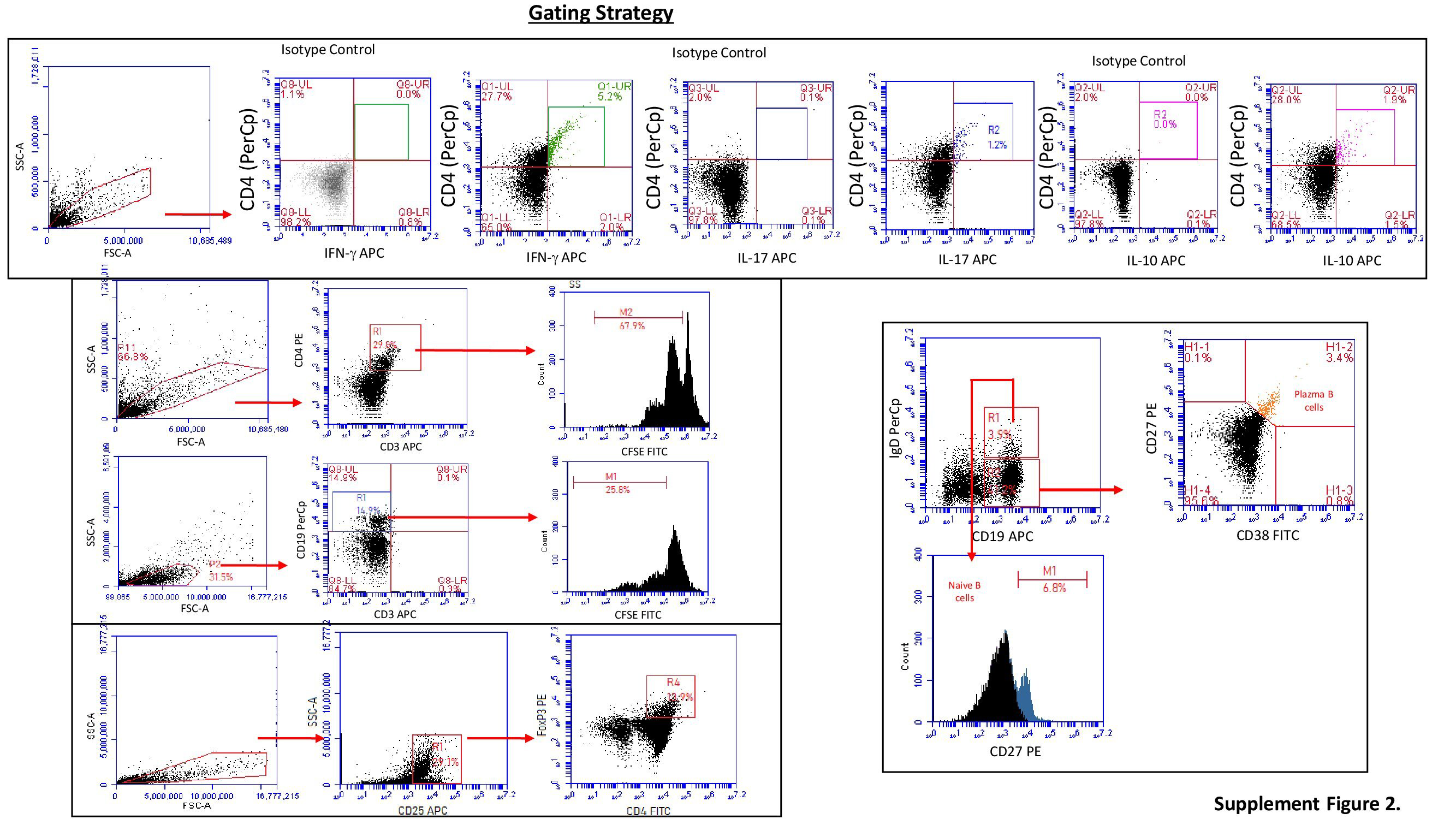

Supplement: S2 Fig — (TIF) [file pone.0266137.s002.tif]

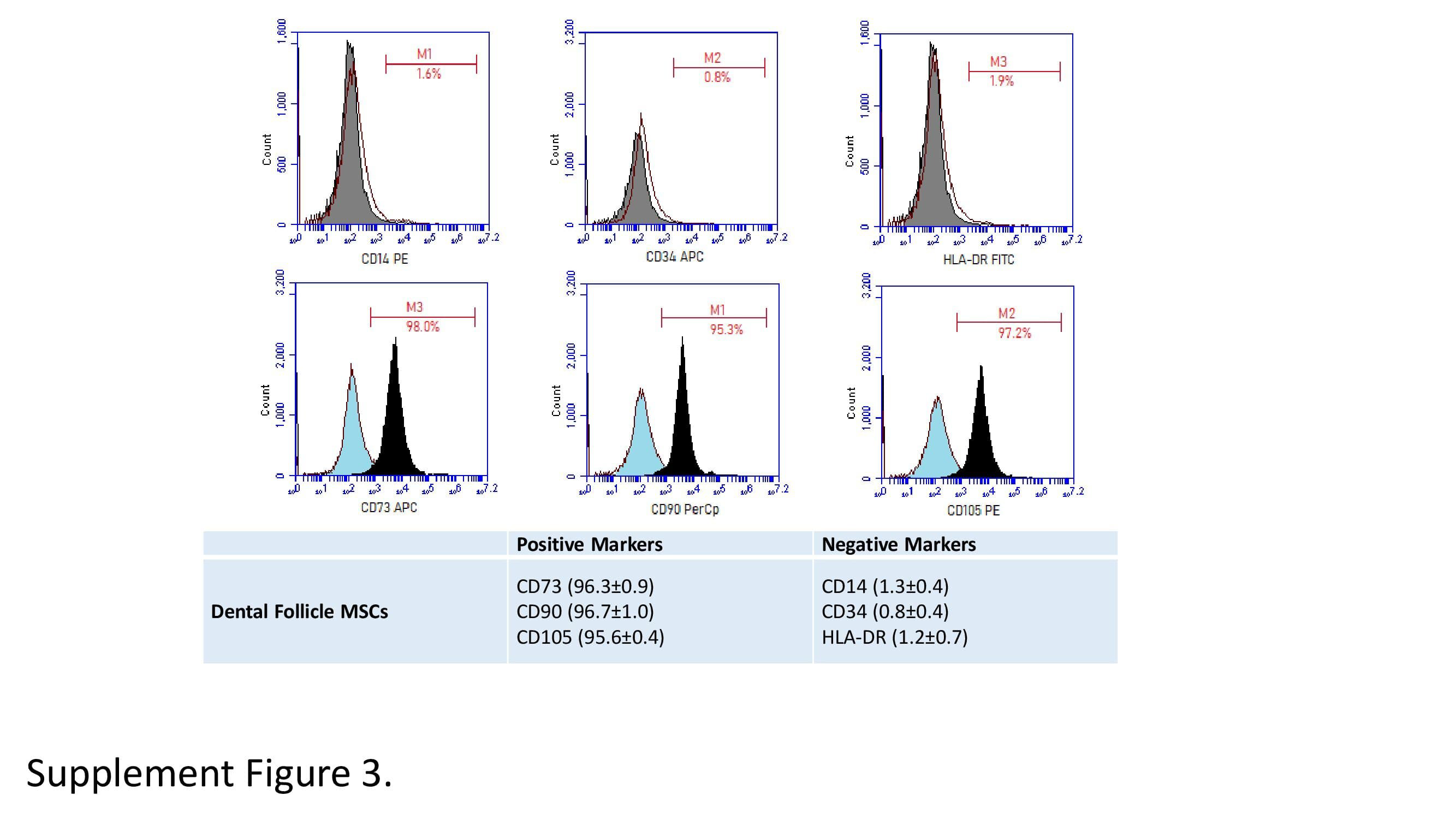

Supplement: S3 Fig — DFMSCs expressed positive markers (CD73, CD90, and CD105) over 95%, and lack the expressions of negative markers (CD14, CD34, and HLA-DR). (TIF) [file pone.0266137.s003.tif]
