# Supplementary material for: Dental follicle mesenchymal stem cells ameliorated glandular dysfunction in Sjögren’s syndrome murine model
Source: PLoS One. 2022 May 5;17(5):e0266137. doi: 10.1371/journal.pone.0266137 (PMC9070867; doi:10.1371/journal.pone.0266137)
Supplement: S1 Raw data — (DOCX) [file pone.0266137.s004.docx]

**Data for: Dental follicle mesenchymal stem cells ameliorated glandular dysfunction in Sjögren’s syndrome murine model**

Deniz GENÇ^1,2^, Osman BULUT^3^, Burcu GÜNAYDIN^4^, Mizgin GÖKSU^5^, Mert DÜZGÜN^5^, Yelda DERE^6^, Serhat SEZGİN^7^, Akın ALADAĞ^7^, Aziz BÜLBÜL^3^

1. Muğla Sıtkı Koçman University, Faculty of Health Sciences, Muğla, TURKEY
2. Muğla Sıtkı Koçman University, Research Laboratories Center, Muğla, TURKEY
3. Muğla Sıtkı Koçman University, Milas Veterinary Medicine Faculty, Muğla, TURKEY
4. Muğla Sıtkı Koçman University, Institute of Health Sciences, Department of Histology and Embryology, Muğla, TURKEY
5. Muğla Sıtkı Koçman University, Faculty of Science, Department of Molecular Biology and Genetics, Muğla, TURKEY
6. Muğla Sıtkı Koçman University, Faculty of Medicine, Department of Pathology, Muğla, TURKEY
7. Muğla Sıtkı Koçman Üniversity, Faculty of Dentistry, Muğla, TURKEY

**Corresponding Author**: Deniz GENÇ, Muğla Sıtkı Koçman University, Faculty of Health Sciences, Muğla, TURKEY. [denizgenc@mu.edu.tr](mailto:denizgenc@mu.edu.tr) Phone: +905337495534.

**The data given below includes the values ​​obtained for statistical analysis**

Data 1. Lacrimal Gland Focus score values

Data 2. Submandibular gland focus score values

Data 3. Flow cytometry analysis values for Lacrimal gland Qdot signaling cells (Mean Fluorescent Intensity)

Data 4. Flow cytometry analysis values for Submandibular gland Qdot signaling cells (Mean Fluorescent Intensity)

Data 5. Flow cytometry analysis values for IL-10 secretion soluble mediators

Data 6: Flow cytometry analysis values for IL-17 secretion soluble mediators

Data 7: Flow cytometry analysis values for IFN-g secretion soluble mediators

Data 8: Flow cytometry analysis values for Lymphocyte proliferation (Mean Fluorescent Intensity)

Data 9: Saliva rate values

Data 10: Tears rate values

Data 11: Flow cytometry analysis values for Saliva IL-10 levels

Data 12: Flow cytometry analysis values for Saliva IL-17 levels

Data 13: Flow cytometry analysis values for Saliva IFN-g levels

Data 14: Flow cytometry analysis values for Tears IL-17 levels

Data 15: Flow cytometry analysis values for Tears IFN-g levels

Data 16: Flow cytometry analysis values for Tears IL-10 levels

Data 17: Flow cytometry analysis values for Naive B lymphocyte ratio (Mean Fluorescent Intensity)

Data 18: Flow cytometry analysis values for Plasma B lymphocyte ratio (Mean Fluorescent Intensity)

Data 1: Lacrimal Gland Focus score

| Control | SS | DFMSCs Lacrimal Injection | DFMSCs Intraperitoneal Injection |
| --- | --- | --- | --- |
| 0 | 5 | 2 | 1 |
| 0 | 3 | 1 | 1 |
| 0 | 3 | 1 | 1 |
| 0 | 4 | 1 | 0 |
| 0 | 3 | 2 | 1 |

Data 2: Submandibular gland focus score

| Control | SS | DFMSCs Submandibular Injection | DFMSCs Intraperitoneal Injection |
| --- | --- | --- | --- |
| 0 | 4 | 2 | 2 |
| 1 | 6 | 3 | 2 |
| 0 | 5 | 1 | 1 |
| 0 | 3 | 0 | 0 |
| 0 | 5 | 2 | 1 |

Data 3: Lacrimal gland Qdot signaling cells

| Lacrimal | Intraperitoneal |
| --- | --- |
| 9 | 1,5 |
| 10,1 | 1,2 |
| 8,9 | 1 |
| 11,2 | 1,1 |
| 10,3 | 0,4 |

Data 4: Submandibular gland Qdot signaling cells

| Submandibular | Intraperitoneal |
| --- | --- |
| 12,7 | 1,8 |
| 11,2 | 1,4 |
| 13,5 | 1 |
| 14,2 | 1,9 |
| 10,3 | 1,2 |

Data 5: IL-10 secretion soluble mediators

| Control | SS | SS+DFMSCs i.p. | SS+DFMSCs Submandibular | SS+DFMSCs Lacrimal |
| --- | --- | --- | --- | --- |
| 28,8 | 14,4 | 24,3 | 11,2 | 11,2 |
| 26,5 | 9,9 | 38,7 | 14,5 | 14,2 |
| 18,9 | 11,2 | 33,5 | 14,2 | 13,1 |
| 21,6 | 18,6 | 41,2 | 11,7 | 10,6 |
| 12,3 | 14,1 | 27,6 | 16,5 | 17,1 |
| 16,1 | 16,5 | 29,2 | 18,8 | 15,3 |
| 16,3 | 9,3 | 31,3 | 17,6 | 12,2 |
| 14,2 | 11,6 | 28,9 | 13,5 | 13,8 |
| 15,6 | 14,1 | 39,5 | 16,1 | 9,6 |

Data 6: IL-17 secretion soluble mediators

| Control | SS | SS+DFMSCs i.p. | SS+DFMSCs Submandibular | SS+DFMSCs Lacrimal |
| --- | --- | --- | --- | --- |
| 202,1 | 487,1 | 197,2 | 501,2 | 487,1 |
| 198,5 | 325,5 | 187,5 | 312,6 | 325,5 |
| 156,2 | 383,2 | 112,7 | 321,5 | 401,2 |
| 296,5 | 271,8 | 131,3 | 271,8 | 271,8 |
| 303,2 | 365,2 | 285,6 | 365,2 | 365,3 |
| 187,1 | 412,3 | 246,5 | 382,6 | 412,3 |
| 176,5 | 321,8 | 217,1 | 411,9 | 321,8 |
| 198,2 | 287,5 | 223,5 | 287,5 | 399,8 |
| 217,3 | 276,9 | 246,9 | 276,9 | 286,5 |

Data 7: IFN-g secretion soluble mediators

| Control | SS | SS+DFMSCs i.p. | SS+DFMSCs Submandibular | SS+DFMSCs Lacrimal |
| --- | --- | --- | --- | --- |
| 25,3 | 38,1 | 17,3 | 38,1 | 41,2 |
| 26,5 | 41,5 | 16,5 | 21,1 | 43,3 |
| 14,2 | 33,3 | 14,8 | 31,2 | 37,5 |
| 26,3 | 31,7 | 13,2 | 33,5 | 22,8 |
| 31,8 | 30,2 | 21,1 | 36,2 | 31,1 |
| 17,2 | 27,5 | 18,6 | 27,5 | 34,5 |
| 19,2 | 56,3 | 14,3 | 41,3 | 38,6 |
| 21,3 | 47,2 | 12,8 | 47,2 | 24,7 |
| 17,9 | 41,6 | 11,7 | 41,6 | 25,5 |

Data 8: Lymphocyte proliferation

| Control | SS | SS+DFMSCs i.p. | SS+DFMSCs Submandibular | SS+DFMSCs Lacrimal |
| --- | --- | --- | --- | --- |
| 10,2 | 61,3 | 33,4 | 58,9 | 71,2 |
| 9,7 | 58,8 | 25,5 | 47,5 | 49,6 |
| 11,5 | 47,5 | 19,7 | 42,2 | 51,1 |
| 13,6 | 46,3 | 24,2 | 31,3 | 36,8 |
| 8,8 | 38,7 | 30,6 | 39,6 | 65,5 |
| 7,4 | 36,5 | 16,3 | 41,1 | 39,3 |
| 10,9 | 41,4 | 17,1 | 40,8 | 46,7 |
| 8,6 | 37,4 | 28,8 | 37,5 | 38,6 |
| 10,9 | 39,8 | 21,6 | 32,3 | 42,5 |

Data 9: Saliva rate

| Control | SS | SS+DFMSCs i.p. | SS+DFMSCs Submandibular | SS+DFMSCs Lacrimal |
| --- | --- | --- | --- | --- |
| 365 | 168 | 187 | 156 | 96 |
| 255 | 142 | 125 | 102 | 121 |
| 301 | 176 | 101 | 96 | 138 |
| 297 | 161 | 134 | 139 | 103 |
| 312 | 137 | 117 | 143 | 141 |
| 312 | 98 | 155 | 121 | 147 |
| 298 | 127 | 147 | 102 | 126 |
| 337 | 171 | 136 | 118 | 113 |
| 321 | 156 | 125 | 111 | 125 |

Data 10: Tears rate

| Control | SS | SS+DFMSCs i.p. | SS+DFMSCs Submandibular | SS+DFMSCs Lacrimal |
| --- | --- | --- | --- | --- |
| 2,9 | 1,2 | 1,4 | 1,2 | 1,2 |
| 3,2 | 1 | 1,4 | 1,2 | 1,8 |
| 2,5 | 1,5 | 1,5 | 1,5 | 1,5 |
| 2,8 | 1,8 | 1,6 | 1,8 | 1,4 |
| 2,7 | 1,6 | 1,6 | 1,9 | 1,6 |
| 2,6 | 1,7 | 1,7 | 1,7 | 1,7 |
| 3 | 2 | 2,2 | 2 | 2,1 |
| 2,5 | 2,1 | 2,1 | 2,3 | 2 |
| 2,9 | 1,1 | 1 | 1,1 | 1,4 |
| 2,8 | 1,4 | 1,3 | 1,3 | 1,4 |

Data 11: Saliva IL-10 levels

| Control | SS | SS+DFMSCs i.p. | SS+DFMSCs Submandibular | SS+DFMSCs Lacrimal |
| --- | --- | --- | --- | --- |
| 10,8 | 10,8 | 12,3 | 17,2 | 10,8 |
| 9,8 | 11,9 | 24,6 | 14,6 | 11,9 |
| 11,2 | 12,2 | 18,5 | 16,5 | 12,2 |
| 12,9 | 9,6 | 12,6 | 11,8 | 11,3 |
| 12,2 | 10,1 | 15,9 | 12,5 | 11,1 |
| 13,6 | 11,2 | 12,2 | 14,9 | 11,2 |
| 15,2 | 9,3 | 16,5 | 11,8 | 9,6 |
| 9,5 | 10,6 | 11,2 | 12,3 | 10,1 |
| 10,8 | 11,5 | 17,8 | 11,1 | 11,3 |

Data 12: Saliva IL-17 levels

| Control | SS | SS+DFMSCs i.p. | SS+DFMSCs Submandibular | SS+DFMSCs Lacrimal |
| --- | --- | --- | --- | --- |
| 2,1 | 17,1 | 12,3 | 11,2 | 21,5 |
| 4,3 | 21,7 | 14,6 | 7,6 | 17,7 |
| 1,8 | 13,8 | 11,3 | 9,2 | 14,8 |
| 3,9 | 17,9 | 12,6 | 11,8 | 18,9 |
| 2,2 | 23,1 | 13,8 | 12,5 | 13,2 |
| 1,3 | 15,6 | 9,2 | 9,3 | 12,5 |
| 1,6 | 16,8 | 11,5 | 10,8 | 21,6 |
| 2,5 | 28,3 | 9,6 | 10,3 | 27,5 |
| 1,8 | 25,6 | 12,2 | 11,1 | 28,7 |

Data 13: Saliva IFN-g levels

| Control | SS | SS+DFMSCs i.p. | SS+DFMSCs Submandibular | SS+DFMSCs Lacrimal |
| --- | --- | --- | --- | --- |
| 10,1 | 12,3 | 9,8 | 6,9 | 12,5 |
| 9,2 | 21,7 | 7,6 | 7,6 | 14,7 |
| 8,9 | 14,8 | 11,3 | 9,2 | 14,8 |
| 8,8 | 17,9 | 12,6 | 9,8 | 18,9 |
| 7,2 | 23,1 | 13,8 | 5,9 | 11,2 |
| 11,3 | 13,6 | 14,2 | 6,3 | 12,5 |
| 7,6 | 16,8 | 12,5 | 7,8 | 14,6 |
| 6,5 | 24,8 | 9,6 | 10,3 | 12,3 |
| 4,8 | 15,6 | 8,9 | 11,1 | 15,5 |

Data 14: Tears IL-17 levels

| Control | SS | SS+DFMSCs i.p. | SS+DFMSCs Submandibular | SS+DFMSCs Lacrimal |
| --- | --- | --- | --- | --- |
| 3,8 | 31,8 | 12,3 | 28,3 | 19,5 |
| 4,1 | 21,7 | 12,6 | 19,6 | 12,1 |
| 1,8 | 21,3 | 11,3 | 25,5 | 11,7 |
| 3,9 | 17,9 | 9,5 | 24,2 | 14,8 |
| 1,6 | 23,1 | 11,8 | 19,1 | 13,2 |
| 6,3 | 15,6 | 15,2 | 21,2 | 12,5 |
| 1,6 | 16,8 | 12,5 | 23,5 | 11,5 |
| 2,5 | 28,3 | 10,9 | 14,9 | 14,2 |
| 7,5 | 25,6 | 12,2 | 12,1 | 15,6 |

Data 15: Tears IFN-g levels

| Control | SS | SS+DFMSCs i.p. | SS+DFMSCs Submandibular | SS+DFMSCs Lacrimal |
| --- | --- | --- | --- | --- |
| 15,2 | 27,3 | 11,8 | 21,3 | 11,1 |
| 9,8 | 21,7 | 12,6 | 27,8 | 16,8 |
| 8,9 | 14,8 | 11,3 | 15,6 | 14,8 |
| 8,8 | 16,8 | 12,6 | 14,2 | 18,9 |
| 10,3 | 17,1 | 13,8 | 11,9 | 10,2 |
| 12,5 | 15,6 | 11,2 | 16,3 | 12,5 |
| 12,6 | 21,3 | 12,5 | 17,1 | 14,6 |
| 12,5 | 24,8 | 9,6 | 15,9 | 10,5 |
| 11,6 | 15,6 | 10,9 | 18,1 | 14,2 |

Data 16: Tears IL-10 levels

| Control | SS | SS+DFMSCs i.p. | SS+DFMSCs Submandibular | SS+DFMSCs Lacrimal |
| --- | --- | --- | --- | --- |
| 12,2 | 10,7 | 14,7 | 10,2 | 16,7 |
| 8,5 | 11,9 | 21,6 | 12,6 | 12,9 |
| 11,6 | 12,2 | 18,5 | 9,5 | 15,5 |
| 14,2 | 9,6 | 16,5 | 10,7 | 12,3 |
| 12,2 | 11,3 | 15,9 | 12,5 | 11,1 |
| 15,3 | 11,2 | 12,2 | 14,9 | 14,1 |
| 12,1 | 10,9 | 16,5 | 13,8 | 8,6 |
| 11,8 | 12,6 | 13,2 | 12,3 | 14,2 |
| 10,9 | 11,5 | 17,8 | 11,1 | 13,1 |

Data 17: Naive B lymphocyte ratio

| Control | SS | SS+DFMSCs i.p. | SS+DFMSCs Submandibular | SS+DFMSCs Lacrimal |
| --- | --- | --- | --- | --- |
| 31,8 | 17,8 | 28,6 | 15,4 | 14,1 |
| 29,6 | 19,7 | 21,2 | 19,8 | 16,9 |
| 27,5 | 21,3 | 24,5 | 14,2 | 21,2 |
| 21,4 | 22,5 | 21,2 | 16,5 | 19,3 |
| 28,6 | 16,4 | 19,5 | 20,2 | 18,7 |
| 30,3 | 17,9 | 26,3 | 21,5 | 15,6 |
| 27,2 | 19,8 | 21,2 | 16,9 | 17,9 |
| 24,4 | 16,3 | 22,5 | 17,1 | 20,8 |
| 27,6 | 23,3 | 26,8 | 18,6 | 19,5 |

Data 18: Plasma B lymphocyte ratio

| Control | SS | SS+DFMSCs i.p. | SS+DFMSCs Submandibular | SS+DFMSCs Lacrimal |
| --- | --- | --- | --- | --- |
| 1,8 | 3,8 | 1,9 | 3,9 | 4,2 |
| 2,2 | 4,1 | 1,7 | 2,8 | 3,9 |
| 2,5 | 6,2 | 2,3 | 3,4 | 2,8 |
| 0,8 | 1,9 | 2,6 | 3,7 | 3,6 |
| 1,3 | 2,2 | 1,8 | 1,6 | 3,1 |
| 1,6 | 2,8 | 0,9 | 3,9 | 3,3 |
| 0,7 | 3,3 | 2,3 | 2,8 | 3,2 |
| 0,4 | 3,6 | 2,9 | 2,5 | 2,8 |
| 0,6 | 2,9 | 1,5 | 3,9 | 3,6 |
