# Supplementary material for: Dental follicle mesenchymal stem cells ameliorated glandular dysfunction in Sjögren’s syndrome murine model
Source: PLoS One. 2022 May 5;17(5):e0266137. doi: 10.1371/journal.pone.0266137 (PMC9070867; doi:10.1371/journal.pone.0266137)
Supplement: Attachment — Submitted filename: Response to Reviewers.docx [file pone.0266137.s005.docx]

28, January, 2022

To The Editor-in-Chief,

We thank the reviewers for their valuable contributions to our study. We revised the manuscript as per reviewers’ comments and explained it in detail below. We changed the figure files in “tif.” file format.

Best regards,

Deniz GENÇ

Corresponding author

**Journal Requirements:**

When submitting your revision, we need you to address these additional requirements.

1. Please ensure that your manuscript meets PLOS ONE's style requirements, including those for file naming. The PLOS ONE style templates can be found at

https://journals.plos.org/plosone/s/file?id=wjVg/PLOSOne_formatting_sample_main_body.pdf and

https://journals.plos.org/plosone/s/file?id=ba62/PLOSOne_formatting_sample_title_authors_affiliations.pdf

***Response****: We edited the manuscript according to the Journal Requirements.*

2. Please provide additional details regarding participant consent. In the ethics statement in the Methods and online submission information, please ensure that you have specified (1) whether consent was informed and (2) what type you obtained (for instance, written or verbal, and if verbal, how it was documented and witnessed). If your study included minors, state whether you obtained consent from parents or guardians. If the need for consent was waived by the ethics committee, please include this information.

***Response****: We included the ethics statements in the Methods section and online submission.*

If you are reporting a retrospective study of medical records or archived samples, please ensure that you have discussed whether all data were fully anonymized before you accessed them and/or whether the IRB or ethics committee waived the requirement for informed consent. If patients provided informed written consent to have data from their medical records used in research, please include this information.

***Response****: The present study is not a retrospective study.*

3. Thank you for stating the following financial disclosure:

“This study is funded by THE SCIENTIFIC AND TECHNOLOGICAL RESEARCH COUNCIL OF TURKEY (TUBITAK) with the Project number of 120S178.”

Please state what role the funders took in the study. If the funders had no role, please state: "The funders had no role in study design, data collection and analysis, decision to publish, or preparation of the manuscript."

If this statement is not correct you must amend it as needed.

Please include this amended Role of Funder statement in your cover letter; we will change the online submission form on your behalf.

***Response****: We thank to the Editorial Board.*

4. Thank you for stating the following in the Acknowledgments Section of your manuscript:

“This study is funded by THE SCIENTIFIC AND TECHNOLOGICAL RESEARCH COUNCIL OF TURKEY (TUBITAK) with the Project number of 120S178. We thank to Rüştü Kuşcu for his skillful assistance.“

“This study is funded by XXXXXXXX with the Project number of 120S178. We thank to XXXXXX for his skillful assistance.”

We note that you have provided additional information within the Acknowledgements Section that is not currently declared in your Funding Statement. Please note that funding information should not appear in the Acknowledgments section or other areas of your manuscript. We will only publish funding information present in the Funding Statement section of the online submission form.

Please remove any funding-related text from the manuscript and let us know how you would like to update your Funding Statement. Currently, your Funding Statement reads as follows:

“This study is funded by THE SCIENTIFIC AND TECHNOLOGICAL RESEARCH COUNCIL OF TURKEY (TUBITAK) with the Project number of 120S178.”

Please include your amended statements within your cover letter; we will change the online submission form on your behalf.

***Response****: We thank to the Editorial Board. We removed the Acknowledgements Section, the funding information has been stated as “This study is funded by THE SCIENTIFIC AND TECHNOLOGICAL RESEARCH COUNCIL OF TURKEY (TUBITAK) with the Project number of 120S178. The funders had no role in study design, data collection and analysis, decision to publish, or preparation of the manuscript.” in the funding information section.*

5. Your ethics statement should only appear in the Methods section of your manuscript. If your ethics statement is written in any section besides the Methods, please move it to the Methods section and delete it from any other section. Please ensure that your ethics statement is included in your manuscript, as the ethics statement entered into the online submission form will not be published alongside your manuscript.

***Response****: The ethics statement is included in the Methods section.*

**Reviewers' comments:**

Reviewer's Responses to Questions

Comments to the Author

1. Is the manuscript technically sound, and do the data support the conclusions?

The manuscript must describe a technically sound piece of scientific research with data that supports the conclusions. Experiments must have been conducted rigorously, with appropriate controls, replication, and sample sizes. The conclusions must be drawn appropriately based on the data presented.

Reviewer #1: Yes

Reviewer #2: Yes

***Response: We thank the reviewers.***

2. Has the statistical analysis been performed appropriately and rigorously?

Reviewer #1: Yes

Reviewer #2: Yes

***Response: We thank the reviewers.***

3. Have the authors made all data underlying the findings in their manuscript fully available?

The PLOS Data policy requires authors to make all data underlying the findings described in their manuscript fully available without restriction, with rare exception (please refer to the Data Availability Statement in the manuscript PDF file). The data should be provided as part of the manuscript or its supporting information, or deposited to a public repository. For example, in addition to summary statistics, the data points behind means, medians and variance measures should be available. If there are restrictions on publicly sharing data—e.g. participant privacy or use of data from a third party—those must be specified.

Reviewer #1: Yes

Reviewer #2: Yes

***Response: We thank the reviewers.***

4. Is the manuscript presented in an intelligible fashion and written in standard English?

PLOS ONE does not copyedit accepted manuscripts, so the language in submitted articles must be clear, correct, and unambiguous. Any typographical or grammatical errors should be corrected at revision, so please note any specific errors here.

Reviewer #1: No

Reviewer #2: Yes

***Response: We thank the reviewers. English editing has been done.***

5. Review Comments to the Author

Please use the space provided to explain your answers to the questions above. You may also include additional comments for the author, including concerns about dual publication, research ethics, or publication ethics. (Please upload your review as an attachment if it exceeds 20,000 characters)

Reviewer #1: In the present study, the Authors investigated the effect of dental follicle (DF)MSCs in regeneration of glandular tissue in Sjögren’s Syndrome (SS) mouse model.

Although the manuscript is good, some issues need to be addressed.

- The figure 7 is out of focus and must be changed.

- Pages 11 and 12: add the name of university and hospital and change the “XXXXXX”.

- The Discussion section is too long (n#6 pages), dispersive and chaotic. Reduce it focusing on results, limits and novelties of work.

***Response 1: Figure 7 was obtained from a fluorescent microscope (Nikon TS-2 FL Trinocular Inverted Microscope 5.9 Megapixel Head DS-Fi3, Japan). The image resolution of the device was 5.9 Megapixels, therefore it cannot be focused by a device further. But, the format of the figures was changed to “tif.” files to increase the resolution of the images.***

***Response 2: The name of the university and hospital has been mentioned in the main text as “Muğla Sıtkı Koçman University”.***

***Response 3: The discussion section has been reduced focusing on the results.***

Reviewer #2: In the present study authors investigated the modulatory and regenerative effects of dental follicle mesenchymal stem cells (DFMSCs) in Sjögren’s Syndrome (SS) mouse model. DFMSCs were injected intraperitoneally, or into submandibular, or lacrimal glands. The immunomodulatory effect was investigated by assessing of intracellular cytokine (IFN-g, IL-17, IL-10) secretion in spleen-derived T helper cells, lymphocyte proliferation and B lymphocyte subsets. The regenerative capability was evaluated by Histologic analysis and flow cytometry of submandibular and lacrimal glands.

The improvement of SS manifestations was observed mainly in systemic injection of DFMSCs, while slight recovery was observed in local injections.

The manuscript is well written and the study is interesting. However, some revisions are needed before the paper can be published:

- In materials and method section, the name of dental hospital is missing.

- Scale bars are missing in Figures 7,8,9

- Histological images need to be improved.

***Response 1: The name of the university and hospital has been mentioned in the main text as “Muğla Sıtkı Koçman University”.***

***Response 2: The scale bars are added in Figures 7, 8, and 9.***

***Response 3: Histological images in Figures 7 and 8 were obtained from a fluorescent microscope (Nikon TS-2 FL Trinocular Inverted Microscope 5.9 Megapixel Head DS-Fi3, Japan). The image resolution of the device was 5.9 Megapixels, therefore it cannot be focused by a device further. We focused the images by changing the file format from “jpeg.” To “tif.”. Histological images in Figure 9 were obtained by a light microscope (Olympus BX46, Japan). The format of the figures was changed to “tif.” files to increase the resolution of the images.***
